# Vulnerable Attachment Style Questionnaire: Preliminary Evidence for a Revised Factor Structure Among Adolescents in Residential Care

**DOI:** 10.3390/children13040551

**Published:** 2026-04-15

**Authors:** Ana Simão, Cátia Martins, Antonia Bifulco, Cristina Nunes

**Affiliations:** 1 University Research Center in Psychology (CUIP), Universidade do Algarve, Campus de Gambelas, 8005-135 Faro, Portugal; 2 CATS—Centre for Abuse, Trauma and Suicide Studies, Middlesex University, The Burroughs, Hendon, London NW4 4BT, UK

**Keywords:** adolescents, attachment, psychological adjustment, psychometric properties, residential care

## Abstract

**Highlights:**

**What are the main findings?**

- The reduced version of the VASQ demonstrated a three-factor structure with acceptable psychometric properties in adolescents in residential care.
- Insecure attachment styles were significantly associated with greater psychological adjustment difficulties, whereas proximity-seeking showed a mixed pattern of associations.

**What are the implications of the main findings?**

- The restructured VASQ shows potential as a brief tool for identifying vulnerable attachment patterns and psychological risk among adolescents in residential care.
- Results underscore the importance of training residential care staff and strengthening caregiver–youth relationships to improve emotional regulation and overall adjustment.

**Abstract:**

**Background/Objectives:** The attachment framework has been used to understand individuals’ development and the emergence of difficulties in specific contexts and developmental stages. The Vulnerable Attachment Style Questionnaire (VASQ) is a brief self-report measure initially designed to assess adult attachment as a vulnerability factor for the development of depression. The present study aimed to examine the factor structure and psychometric properties of the VASQ in a sample of adolescents living in residential care and to provide preliminary evidence for a revised version of the instrument in this population. **Methods:** A total of 438 youths, aged 12 to 18 years (M = 15.34), completed the questionnaire. Data were randomly split to conduct independent exploratory and confirmatory factor analyses. **Results:** A three-factor model, consisting of two types of insecurity (ambivalent and avoidant) and a proximity-seeking dimension, emerged as the best-fitting solution. This revised structure involved removing several items while maintaining acceptable psychometric properties and meaningful associations with psychological adjustment. Concurrent validity was assessed by examining youth psychological adjustment, and positive correlations emerged as expected. **Conclusions:** The findings provide preliminary support for a shortened, restructured version of the VASQ for adolescents in residential care. Rather than confirming the original factorial structure, the results highlight the need to consider population-specific adaptations of the instrument. This version may have potential utility in clinical or intervention contexts and underscores the need to train institutional workers to develop competencies tailored to this population.

## 1. Introduction

Adolescents in residential care settings (RCSs) often face significant developmental challenges stemming from early adversity, instability, and disruptions in caregiving relationships. These experiences are consistently linked to greater emotional, behavioral, and social difficulties than those of peers raised in family environments [1,2,3]. In Portugal, most children under state protection are placed in residential care, and about 25% enter care due to behavioral problems. This underscores the importance of understanding this population’s developmental needs [4].

Attachment theory provides a relevant framework for examining these challenges, highlighting how early relationships shape emotional regulation, interpersonal functioning, and psychological adjustment [5,6,7]. Disruptions in attachment—particularly those involving inconsistent or multiple caregivers—are linked to increased vulnerability to psychopathology and maladaptive developmental trajectories [5,6,8,9,10]. This is especially pertinent in RCSs, where caregiving environments are often marked by high staff turnover and limited opportunities for stable, individualized relationships [11,12,13,14].

Despite the importance of attachment processes in this context, assessing them in adolescents—particularly those in institutional care—remains complex. Existing measures vary in format and focus, including observational methods, interviews, and self-report questionnaires. However, many of these instruments are neither brief nor easy to administer and interpret, and few have been specifically developed for use with institutionalized youth [12]. Furthermore, research on the reliability and validity of self-report attachment measures in this population remains limited [12], underscoring the need for further investigation.

The Relationship with Significant Figures Questionnaire [15] assesses adolescents’ relationships with significant figures through 28 items across two dimensions (teachers/school staff and institutional staff) on a 6-point Likert scale. It has demonstrated adequate reliability (α = 0.88) [16]. However, this instrument does not capture behavioral components of attachment or adequately address associations between attachment vulnerabilities and adolescent depression [17], which is prevalent in this population [18]. Accordingly, some researchers have emphasized the need for tools that can effectively assess vulnerable attachment and identify individuals at risk of psychopathology, particularly depression [17,19].

The Vulnerable Attachment Style Questionnaire (VASQ) is a brief self-report instrument developed to assess attachment-related vulnerability, particularly in relation to depression [19]. It yields a total vulnerable attachment score and subscale scores reflecting insecurity and proximity-seeking patterns. Previous studies have reported acceptable psychometric properties, including internal consistency and test–retest reliability, as well as meaningful associations with psychological outcomes, suggesting that the VASQ is a promising assessment tool [19,20]. However, validation studies have primarily been conducted in community samples, and its applicability to adolescents in residential care remains underexplored [21]. The VASQ was designed to assess attachment-related behaviors, emotions, and attitudes and may therefore be particularly useful for capturing variability within the residential care experience [22]. Although it was not originally developed for institutionalized populations, it has shown promising results in this context [22]. In Portugal, several studies have employed the VASQ among adolescents in residential care [23,24,25]; however, formal validation in the Portuguese context remains lacking. Moreover, a comprehensive and consistent examination of the scale’s factorial structure in this population is warranted.

### Current Study

Although the VASQ has been validated, prior work has primarily used community-based samples. As such, it does not adequately account for the specific contextual and relational characteristics of adolescents in RCSs—such as disrupted attachment histories, multiple caregiving relationships, and institutional environments—that may influence the structure and expression of attachment dimensions [12,22]. Therefore, it is important to examine whether the original factorial structure of the VASQ is appropriate for this population or whether alternative configurations provide a better fit.

The original two-factor structure captures distinct yet related dimensions of attachment-related difficulties, typically conceptualized as insecurity and proximity-seeking or avoidance patterns [19]. This distinction may be particularly relevant for adolescents in residential care, who often have disrupted attachment histories, multiple caregiving relationships, and relational instability. These experiences are linked to heightened emotional insecurity and difficulty forming and maintaining close relationships. Accordingly, a two-factor model aligns with the relational and emotional profiles commonly observed in this population. Nevertheless, given the unique characteristics of institutionalized youth, it remains essential to empirically test whether this structure holds in this context.

The present study aims to examine the factor structure and psychometric properties of the VASQ among adolescents in residential care and to explore its potential utility in this population. The primary objective is to establish the VASQ as a self-report instrument capable of identifying adolescents at risk of maladjustment. This will be achieved by examining its factor structure, assessing its reliability, evaluating its concurrent validity with external variables, and exploring its clinical utility in relation to psychological adjustment. Specifically, this study seeks to: (1) examine the factor structure of the VASQ; (2) test the adequacy of the original two-factor model; (3) assess the internal consistency of the scale; and (4) evaluate its concurrent validity and clinical utility through associations with psychological adjustment.

Based on prior literature, the following hypotheses were formulated:

**H1.**  *A two-factor structure of the VASQ is expected, though alternative factor solutions will also be explored given the target population’s characteristics;*

**H2.**  *The two-factor model is expected to fit the data acceptably; however, alternative models will be examined to identify the best-fitting structure;*

**H3.**  *The VASQ is expected to demonstrate adequate internal consistency;*

**H4.**  *The VASQ is expected to show evidence of construct validity through its associations with psychological adjustment.*

## 2. Materials and Methods

### 2.1. Participants

The sample comprised 438 adolescents from 46 Portuguese RCSs, including 195 boys (44.5%) and 243 girls (55.5%), aged 12 to 18 years (M = 15.34, SD = 1.76). Participants were evenly distributed across age groups (12–15 years: *n* = 217, 49.5%; 16–18 years: *n* = 221, 50.5%). The 12–24 age group represents the largest proportion of individuals in Portuguese residential care institutions [4]. On average, participants had been living in their current RCS for 41 months (SD = 43; range = 1–204 months). Regarding educational level, the majority of participants (57%) were attending middle school. Most participants (93%) had siblings; however, only 31% were placed in the same institution.

### 2.2. Measures

#### 2.2.1. Vulnerable Attachment Style Questionnaire (VASQ [19,24])

The VASQ is a brief self-report instrument designed to assess adult attachment styles as a vulnerability factor for depression. It comprises 22 items that evaluate attachment-related behaviors, emotions, and attitudes and was validated against the Attachment Style Interview [26,27]. Participants rate each item on a 5-point Likert scale based on how they generally feel, rather than their current emotional state. Items 14 and 15 are positively worded and require reverse scoring to ensure consistency in the scale’s direction. The VASQ provides a total score of vulnerable attachment, as well as two subscale scores reflecting “Insecurity” and “Proximity-seeking” attachment styles. Higher total scores indicate greater attachment vulnerability, while higher subscale scores reflect greater insecurity or proximity-seeking tendencies. In addition, the scoring procedure allows the identification of disorganized attachment patterns using established cutoff scores that indicate dysfunctional levels. Low scores on both the mistrustful avoidance and anxious proximity-seeking dimensions indicate a secure attachment style [22].

The Portuguese version of the VASQ was developed using a forward–backward translation procedure conducted by two translators with expertise in psychological research. Cultural adaptation was carefully addressed, with particular attention to clarity, the use of commonly understood language, and conceptual equivalence [24]. Established cross-cultural adaptation guidelines were followed to minimize item bias and differential item functioning [28]. Any discrepancies between translations were resolved through discussion until full semantic equivalence between the original and Portuguese versions was achieved.

#### 2.2.2. Strengths and Difficulties Questionnaire (SDQ [29,30])

The SDQ is a 25-item self-report instrument designed to assess socio-emotional difficulties in children and adolescents. Items are rated on a 3-point Likert-type scale. The instrument comprises five subscales, and a total difficulties score can be computed to reflect overall mental health functioning [29,31]. In the original version, internal consistency coefficients ranged from 0.41 to 0.80 [32]. In the Portuguese adaptation, Cronbach’s alpha values ranged from 0.43 to 0.61 [30]. In the present study, internal consistency coefficients were as follows: emotional symptoms α = 0.68, behavioral problems α = 0.57, hyperactivity/inattention α = 0.66, peer relationship problems α = 0.51, prosocial behaviors α = 0.78, and total difficulties α = 0.78.

### 2.3. Procedures

#### 2.3.1. Data Collection

Ethical approval for the study was obtained from the Ethics Committee of the University of Algarve (CEUAlg Pn° 110/2023). All procedures were conducted in accordance with the ethical standards of the 1964 Declaration of Helsinki and its subsequent amendments, or with comparable ethical guidelines. Authorization to validate the VASQ in the Portuguese residential care context was also obtained from the authors of the original instrument and its Portuguese translation.

A pilot study with 15 adolescents assessed the clarity and comprehensibility of the translated version. Participants were invited to report any difficulties understanding or interpreting the items during administration. No significant comprehension issues were identified, so no item-level revisions were required. No formal expert review was conducted at this stage.

A convenience sample was recruited from 46 RCSs across mainland Portugal, the Azores, and Madeira archipelagos, all of which agreed to participate. Institutions were invited to take part in the study; however, inclusion depended on their availability and willingness to collaborate, resulting in a non-random sample. Although the sample is relatively large and provides valuable data, it may not be fully representative of all adolescents in residential care because institutions that declined participation were not included.

Data collection was conducted primarily in person by the first author whenever feasible. When geographical constraints prevented in-person administration, questionnaires were mailed and administered by a designated professional within the institution. Instructions and clarifications were provided via email and reiterated in writing at the time of questionnaire distribution.

Participants were eligible if they were between 12 and 18 years of age, fluent in Portuguese, and free from medical conditions that could interfere with participation. Adolescents with cognitive impairments, as identified by institutional professionals, were excluded. These determinations were based on staff members’ routine observations and knowledge of the adolescents, as no standardized cognitive assessment was administered across institutions.

Eligible participants were informed of the study’s purpose and invited to participate voluntarily. To ensure voluntariness in the institutional context, it was clearly stated that participation was entirely optional and that refusal or withdrawal would not result in any negative consequences. Participants were also assured that their responses would remain confidential and would not be shared with caregivers or institutional staff in an identifiable form. To minimize any perception of coercion, it was explicitly clarified that participation would not affect their care or status within the institution.

All participants provided written informed consent. For adolescents under 16, assent was obtained in addition to informed consent from a legal guardian. When direct guardian consent was unavailable, the institution’s technical director provided consent in accordance with national child protection legislation and standard procedures governing RCSs in Portugal. These procedures permit the designated institutional representative to act in the child’s best interests when legal guardianship is delegated or cannot be exercised promptly. This approach aligns with established ethical guidelines for research involving minors in protective care contexts.

Adolescents completed an anonymous, structured self-report questionnaire at their residential care institution. In addition, directors of the participating institutions completed structured questionnaires to provide information on organizational characteristics.

#### 2.3.2. Data Analysis

Data analyses were conducted using IBM SPSS Statistics 30.0 [33] and Jamovi (version 2.6) [34,35,36].

To examine the factorial structure of the VASQ, the total sample was randomly split into two subsamples. One subsample was used for Exploratory Factor Analysis (EFA), and the other for Confirmatory Factor Analysis (CFA). This approach is commonly used to reduce the risk of overfitting and to provide independent validation of the factor structure. The two subsamples were compared on key sociodemographic variables, including age, gender, and length of stay in residential care. No significant differences were observed, indicating their comparability.

A random subsample of 150 participants was used for the EFA. Varimax rotation was initially applied to enhance interpretability, assuming factor independence, consistent with previous recommendations [37,38]. Given expected correlations among psychological constructs, an oblique rotation (Promax) was also examined. Results were consistent across rotation methods, supporting the stability of the factor solution. Skewness (S) and kurtosis (K) were assessed, with values exceeding 3.0 for skewness and 8.0 for kurtosis considered indicative of serious deviations from normality [39].

Factor retention was based on convergence between visual inspection of the scree plot and parallel analysis using Monte Carlo simulation (2000 replicates), with eigenvalues greater than 1 considered. The Kaiser–Meyer–Olkin (KMO) measure was used to assess sampling adequacy. Communalities were examined for all items, and item retention was guided by established psychometric criteria. Specifically, items were retained if they demonstrated primary factor loadings ≥ 0.40 and did not exhibit substantial cross-loadings (i.e., loadings ≥ 0.40 on more than one factor). Items failing to meet these criteria were considered for removal. Items with low loadings (<0.40) were excluded due to insufficient association with any factor, whereas items with cross-loadings were removed because they compromised the interpretability and factorial purity of the solution. In addition to statistical criteria, theoretical coherence and conceptual interpretability were considered in decisions about item retention.

The best-fitting factor solutions from the EFA were subsequently tested via CFA using the remaining subsample (*n* = 288).

Given the ordinal nature of the Likert-scale data, CFA was conducted using robust Maximum Likelihood estimation (MLR), which is robust to non-normality [40,41]. Model fit was evaluated using multiple goodness-of-fit indices, including the chi-square to degrees of freedom ratio (χ^2^/*df*), Comparative Fit Index (CFI), Tucker–Lewis Index (TLI), and Root Mean Square Error of Approximation (RMSEA) [42,43]. Acceptable model fit was defined as CFI and TLI ≥ 0.90 and RMSEA ≤ 0.10.

The chi-square statistic evaluates the discrepancy between the observed covariance matrix and the one implied by the model. However, because it is sensitive to sample size, significant values are common in large samples, even when the model fit is acceptable [39]. Therefore, additional fit indices (CFI, TLI, RMSEA) were used to provide a more comprehensive evaluation.

CFA was conducted using the original item set and a categorical correlation matrix. Items with standardized factor loadings below 0.40 were removed. No modification indices were used to improve model fit, preserving the theoretical integrity of the measurement model.

Pearson correlations were computed to examine associations among variables. Correlations were interpreted as small (<0.20), moderate (0.20–0.50), or large (>0.50) [39]. Mean inter-item correlations were considered adequate if they ranged from 0.15 to 0.50. Corrected item-total correlations were considered acceptable if they were above 0.20, and Cronbach’s alpha coefficients were considered adequate if they were ≥0.70 [39].

Additional analyses examined associations between VASQ dimensions and psychological adjustment. Receiver operating characteristic (ROC) curve analysis and cross-tabulations were used to assess classification accuracy. Sensitivity and specificity were evaluated, and the area under the curve (AUC) was calculated to determine optimal cutoff values for each VASQ dimension. Chi-square (χ^2^) statistics, Cramér’s V, and *odds ratios* (OR) were computed to assess the strength of associations between VASQ dimensions and SDQ subscales, as well as the total difficulties score [44,45].

For the chi-square analyses, SDQ outcomes were dichotomized into clinical and non-clinical categories, with the non-clinical category serving as the reference group. Accordingly, an OR below 1.00 indicates a higher likelihood of clinically significant difficulties.

## 3. Results

### 3.1. Exploratory Factor Analysis of the VASQ

Table 1 presents the means, standard deviations, skewness, and kurtosis for the VASQ items. Most items had skewness and kurtosis within acceptable ranges, with no evidence of severe deviations from normality, indicating that the assumption of approximate normality was met. Mean scores showed adequate variability across items (M_Range_ = 2.35–4.05).

Corrected item-total correlations for the Portuguese version of the VASQ were generally acceptable. However, items 2, 15, and 21 showed lower correlations, suggesting they may not contribute meaningfully to the assessment of the attachment construct. Alpha values did not improve substantially upon deletion of any item.

Factor solutions with two to four factors were examined. This decision was based on converging evidence from multiple sources: visual inspection of the scree plot, which indicated an inflection point at the third component; examination of the component matrix; and results from parallel analysis using Monte Carlo simulation.

Eigenvalues greater than 1 were observed, and sampling adequacy was supported by the Kaiser–Meyer–Olkin index (KMO = 0.78). The initial three-factor solution revealed the expected dimensions of insecurity and proximity-seeking. However, several items exhibited substantial cross-loadings despite factor loadings above 0.40. Specifically, items 3, 5, and 19 (originally associated with insecurity) and items 11, 13, and 16 (from the proximity-seeking dimension) loaded on multiple factors. Given the ambiguity in their factorial allocation, these items were excluded from further analyses.

Table 2 presents the EFA results for the VASQ, including factor loadings, communalities, and explained variance. After item removal, the three-factor solution showed acceptable internal consistency: insecurity-ambivalent (α = 0.75; 23% of explained variance), insecurity-avoidant (α = 0.61; 12%), and proximity-seeking (α = 0.67; 9%). All retained items showed satisfactory communalities and factor loadings above 0.40, indicating a stable and interpretable structure.

Contrary to the initial hypothesis of a two-factor model, the findings supported a three-factor solution, indicating differentiation within the insecurity dimension in this population. This model was retained for further analysis because of its empirical adequacy, theoretical interpretability, and convergence across extraction criteria, including parallel analysis (see Table 2).

### 3.2. Confirmatory Factor Analysis of the VASQ

An initial CFA was conducted to test the original two-factor structure [19]; however, model fit indices indicated poor fit, with all values falling outside recommended thresholds. Based on the EFA results, a revised three-factor model was subsequently tested using CFA with robust maximum-likelihood estimation in the second subsample (*n* = 288).

The CFA initially included 16 items; however, items 12, 15, and 21 were removed due to low standardized factor loadings (<0.40). The final model comprised 13 items and demonstrated adequate fit to the data (see Table 3). Factor loadings and communalities supported the model’s adequacy, and fit indices indicated improvement relative to previously reported models [19,21], suggesting superior performance in this sample.

Internal consistency indices for the three subscales are presented in Table 4, along with mean inter-item correlations and corrected item-total correlations. All subscales demonstrated acceptable reliability, with item-total correlations exceeding 0.36. The insecure-ambivalent subscale showed satisfactory internal consistency, whereas the insecure-avoidant and proximity-seeking subscales showed borderline but acceptable values for exploratory research.

**Figure 1:**
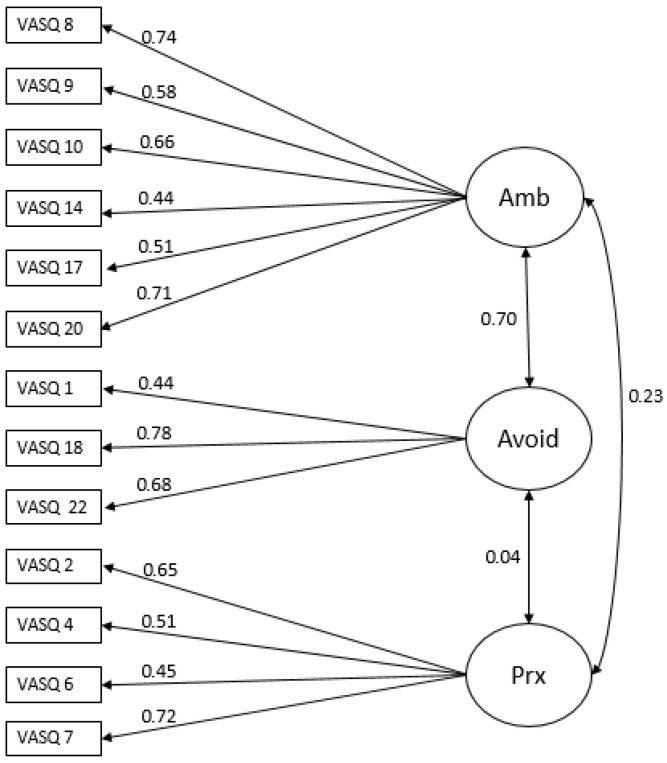
Path diagram of the confirmatory factor analysis.

Figure 1 presents the path diagram of the final model, including standardized factor loadings and covariances among latent dimensions.

### 3.3. Concurrent Validity with External Variables

To assess concurrent validity, Pearson correlations were computed between VASQ dimensions and psychological adjustment, as measured by the Strengths and Difficulties Questionnaire (SDQ). As shown in Table 5, all VASQ dimensions were positively and significantly associated with SDQ subscales and the total difficulties score. Correlations ranged from weak to strong, with the highest associations observed for the insecurity-ambivalent dimension, indicating a stronger link to psychological maladjustment. Overall, these findings support the concurrent validity of the VASQ.

Receiver operating characteristic (ROC) analyses were conducted to assess the ability of VASQ dimensions to discriminate adolescents with clinically significant difficulties across SDQ domains (see Table 6). These findings align with established SDQ practice, which uses cutoff points to categorize risk levels and identify clinical cases. The results provide preliminary evidence that a shortened three-factor version of the VASQ may be useful for at-risk adolescent populations.

The insecurity-ambivalent dimension demonstrates the strongest discriminative performance, particularly for total difficulties (AUC = 0.791, 95% CI [0.745–0.837], *p* < 0.001), with balanced sensitivity (0.706) and specificity (0.725), yielding a Youden index of 0.431. It also shows moderate accuracy for emotional symptoms (AUC = 0.707, *p* < 0.001) and behavioral problems (AUC = 0.689, *p* < 0.001).

The insecurity-avoidant dimension showed modest discriminative ability, with significant but lower AUCs for emotional symptoms (AUC = 0.646, *p* < 0.001), peer problems (AUC = 0.644, *p* < 0.001), and total difficulties (AUC = 0.618, *p* < 0.001). Its discriminative ability for behavioral problems and hyperactivity was limited and non-significant.

In contrast, the proximity-seeking dimension showed weak discriminative power. Although some associations reached statistical significance (e.g., emotional symptoms: AUC = 0.618, *p* = 0.001), sensitivity, specificity, and the Youden index were low, indicating limited clinical utility. Several outcomes showed non-significant or near-chance discrimination.

Cutoff points were determined using the maximum Youden index. However, for several models—particularly those involving the proximity-seeking dimension—low Youden values suggest limited practical applicability.

These findings indicate that the ambivalent dimension of attachment is the most relevant for identifying adolescents at risk of psychological difficulties in residential care, whereas proximity-seeking appears to have limited discriminative power.

Table 6 summarizes the cutoff values and associated indices for each VASQ dimension in relation to the SDQ domains. Only statistically significant and clinically meaningful ROC results are presented; full results are provided in Appendix A.

Finally, Table 7 presents associations between VASQ dimensions and clinically significant difficulties across SDQ domains. Given the coding of the outcome variables, an OR below 1.00 indicates a higher likelihood of being in the clinical range. These analyses further support the insecurity-ambivalent dimension as the most relevant predictor of psychological maladjustment in this population.

Table 7 shows that the insecure-ambivalent dimension exhibited the most consistent and robust pattern of associations. Significant relationships were observed with emotional symptoms (χ^2^(1) = 23.87, *p* < 0.001, OR = 0.32), behavioral problems (χ^2^(1) = 26.41, *p* < 0.001, OR = 0.31), hyperactivity (χ^2^(1) = 12.81, *p* < 0.001, OR = 0.43), peer problems (χ^2^(1) = 5.75, *p* = 0.017, OR = 0.52), and total difficulties (χ^2^(1) = 72.10, *p* < 0.001, OR = 0.16). Effect sizes ranged from small to large, with the strongest association for total difficulties. Overall, these results indicate that higher levels of ambivalent attachment are associated with a substantially increased likelihood of clinically significant difficulties across all assessed domains.

The insecure-avoidant dimension also showed significant associations with emotional symptoms (χ^2^(1) = 16.75, *p* < 0.001, OR = 0.39), peer problems (χ^2^(1) = 11.87, *p* < 0.001, OR = 0.39, and total difficulties (χ^2^(1) = 15.52, *p* < 0.001, OR = 0.43), although effect sizes were consistently smaller. No significant associations were found for behavioral problems, hyperactivity, or prosocial behavior. These findings suggest that higher levels of avoidant attachment are associated with an increased likelihood of clinically significant difficulties, particularly in the emotional and interpersonal domains.

The proximity-seeking dimension showed a less consistent pattern. Significant associations were observed for emotional symptoms (χ^2^(1) = 15.28, *p* < 0.001, OR = 0.39), hyperactivity (χ^2^(1) = 5.40, *p* = 0.020, OR = 0.56), prosocial behavior (χ^2^(1) = 7.84, *p* = 0.005, OR = 3.33), and total difficulties (χ^2^(1) = 5.46, *p* = 0.019, OR = 0.59). However, effect sizes were small, and associations with behavioral and peer problems were non-significant. Overall, higher proximity-seeking was associated with an increased likelihood of clinically significant difficulties in some domains (as indicated by OR < 1), although the association with prosocial behavior (OR = 3.33) suggests a different pattern, with higher proximity-seeking linked to a greater likelihood of non-clinical functioning in this domain.

These findings indicate that the ambivalent attachment dimension is most strongly associated with psychological difficulties, followed by the avoidant dimension, whereas proximity-seeking shows weaker and less consistent associations. This pattern aligns with the ROC analyses, which likewise identified the ambivalent dimension as the most robust discriminator of psychological risk among adolescents in residential care.

Overall, the findings support a revised three-factor structure of the VASQ in this sample and offer preliminary evidence of its psychometric adequacy among adolescents in residential care.

## 4. Discussion

The present study aimed to examine the factorial structure, reliability, and validity of the VASQ among adolescents living in RCSs. Overall, the findings provide partial support for the proposed hypotheses and contribute to a more refined understanding of attachment assessment in high-risk populations.

Contrary to expectations, the original two-factor structure of the VASQ [19] was not supported. Instead, both exploratory and confirmatory analyses converged on a three-factor solution, distinguishing proximity-seeking, insecure-ambivalent, and insecure-avoidant dimensions. This finding suggests that attachment-related constructs among adolescents in RCSs may be more differentiated than originally conceptualized. In particular, the division of the insecurity dimension into ambivalent and avoidant components indicates distinct relational patterns within this population. Therefore, rather than confirming the original factorial structure, the present results suggest an empirical reformulation of the VASQ and highlight the need to consider population-specific attachment configurations.

These results support the view that attachment manifestations vary across developmental stages and contexts, particularly in unstable environments with disrupted caregiving relationships. Rather than indicating a limitation, the divergence from the original structure may suggest that the initial model does not fully capture the complexity of attachment experiences among institutionalized youth. Accordingly, the findings underscore the importance of re-evaluating established measurement models when applied to high-risk populations.

The revised three-factor structure for the VASQ retains similarities with the original version [19], but two notable differences emerged. First, item 14—originally included in the proximity-seeking subscale [19]—loaded onto the insecurity domain in our study. Second, eight items were removed from the total scale to achieve a good model fit because of weak or cross-loadings. The resulting three-factor solution provides acceptable psychometric properties, including adequate reliability, thereby supporting the second and third hypotheses.

Although several items were removed because of weak or cross-loadings, the resulting model showed improved fit and interpretability. These modifications likely reflect both methodological refinement and contextual influences, including relational instability, multiple caregivers, and developmental changes associated with adolescence.

Some items originally designed for adults or the general population may not fully capture the relational dynamics of this group. For example, items related to autonomy or reliance on others may be interpreted differently in RCSs, where opportunities for independent decision-making or for stable trust relationships may be limited.

Although each of the three subscales now contains fewer items than in previous versions [19,21], the present study demonstrated acceptable reliability across all subscales.

Regarding validity, the results supported the expected associations between attachment insecurity and psychological maladjustment. Previous research suggests that trauma exposure may affect child well-being through mechanisms involving insecure attachment. Studies with foster youth have also shown that psychological adjustment is linked to the quality of caregiver relationships [1]. Adolescents living in RCSs are exposed to adverse life circumstances that can increase the likelihood of reporting insecure or disorganized attachment styles [28], and these styles are closely linked to psychological adjustment difficulties [46]. Research consistently shows that these adolescents are more likely to display insecure, particularly disorganized, attachments than their peers in community- or family-based settings, which, in turn, leads to greater challenges in emotion regulation and interpersonal relationships [25,47]. Additionally, youth in RCSs tend to exhibit higher rates of adjustment problems than their peers in the general population [48]. Those with a history of secure attachments are generally better equipped to adapt to new environments, whereas individuals with insecure attachment histories are at greater risk for adjustment difficulties and poorer mental health outcomes [46,49]. The quality of relationships with caregivers in RCSs is a critical factor, as sensitive and responsive caregiving can help mitigate some of these risks and foster emotional security [23,47].

The present sample revealed distinct associations between attachment styles and psychological difficulties, confirming our fourth hypothesis. Across all analyses, the insecure-ambivalent dimension emerged as the most robust correlate of psychological difficulties, showing consistent associations with emotional, behavioral, and overall difficulties. Chi-square analyses further indicated that higher levels of ambivalent attachment were associated with an increased likelihood of clinically significant difficulties across domains, reinforcing its role as a particularly salient risk factor in residential care contexts [25], possibly reflecting heightened sensitivity to relational inconsistency and fear of abandonment.

The insecure-avoidant dimension showed more modest yet still significant associations, particularly in the emotional and peer domains. Consistent with the results in Table 7, higher levels of avoidant attachment were associated with a greater likelihood of clinically significant difficulties, though the effect sizes were smaller. This pattern aligns with theoretical accounts suggesting that avoidant strategies involve emotional distancing and reduced help-seeking, which may contribute to interpersonal and emotional difficulties.

In contrast, the proximity-seeking dimension showed a weaker and less consistent association. Although higher levels of proximity-seeking were associated with an increased likelihood of clinically significant difficulties in some domains, these effects were generally small and less consistent across outcomes. Notably, the association with prosocial behavior showed the opposite pattern, indicating that greater proximity-seeking was linked to a higher likelihood of non-clinical functioning in this domain. This dual pattern supports the interpretation of proximity-seeking as a more complex and context-dependent dimension, potentially reflecting both adaptive efforts to seek support and underlying relational vulnerability. This attachment style could be interpreted as a protective factor that reduces the risk of adjustment problems and may represent a dimension of interpersonal sensitivity. The desire for connection may buffer against social isolation, which is associated with better emotional regulation in group settings and enhanced resilience [50,51]. This suggests an insecure attachment pattern that nonetheless presents some adaptive and resilient characteristics, which may indicate that such difficulties are less likely to occur in these young people [44,45].

Although it is not a fully secure attachment style, the proximity-seeking style tends to show less insecurity than the other two and allows for some autonomy. Proximity-seeking can also coexist with insecure attachment patterns, leading to hypervigilance about relational threats [52], intense emotional reactions when proximity needs are unmet, and difficulties with self-soothing, which may contribute to hyperactivity or emotional outbursts [53]. Our findings indicate a correlation between the proximity-seeking dimension and the insecure-ambivalent attachment style (see Figure 1), as well as between proximity-seeking and emotional problems.

Taken together, the convergence of factor-analytic, correlational, ROC, and categorical analyses strengthens confidence in the proposed three-factor structure and underscores the central role of ambivalent attachment in understanding adolescents’ psychological risk in residential care.

Theoretically, these results underscore the importance of treating attachment as a multidimensional construct, particularly in high-risk and non-normative populations. Adolescents in residential care often develop internal working models shaped by early adversity, leading to expectations of inconsistency or unavailability in relationships [5]. These models may manifest differently across attachment dimensions, with ambivalent patterns reflecting heightened emotional reactivity and avoidant patterns reflecting relational withdrawal [23,24]. Frequent placement changes and disruptions to emotional bonds further undermine socioemotional development [42].

If caregivers inconsistently meet a child’s needs, proximity-seeking may become associated with fear of abandonment, even amid a desire for closeness, resulting in a paradoxical “autonomy–proximity imbalance” [53] marked by craving connection while doubting its reliability. Thus, proximity-seeking can reflect secure attachment when caregivers respond consistently—leading to healthy social skills—or insecure attachment when responses are erratic, triggering anxiety and emotional volatility [50]. In supportive environments, this style promotes collaboration [51,52]; however, in unstable settings such as RCSs, it can exacerbate distress due to unmet expectations.

Regarding the positive and significant correlation between the proximity-seeking dimension and psychological adjustment, it is noteworthy that this association was weaker than those observed for the other two dimensions. This may be attributed to adolescents in these contexts’ tendency to report more insecure attachments. Additionally, those who experience security may attribute it to being removed from situations of neglect or deprivation, thus providing an opportunity to establish secure connections with new significant others. This context enables them to perceive their new environment as enhancing feelings of safety [43] and fostering proximity-seeking behaviors. In this sense, secure base relationships may facilitate proximity-seeking, which, in turn, promotes psychosocial adaptation by providing opportunities for care in this setting. Similarly, individuals with secure base relationships tend to exhibit fewer mental health issues and more positive relational experiences [47], thereby reducing barriers to help-seeking.

In addition to introducing a new self-report assessment and scoring method that may benefit researchers, institutional professionals, and clinicians, the VASQ supports a model of attachment style and disorder [19] that appears particularly relevant in institutional settings. ROC analyses further support the scale’s clinical utility and discriminative ability in identifying adolescents with higher levels of psychological difficulties.

Although the CFA indicated an acceptable fit for the proposed three-factor model, these findings should be interpreted cautiously. The final structure was obtained after item removal and model refinement, which may capitalize on sample-specific characteristics and limit generalizability. In addition, two subscales showed only borderline internal consistency, indicating that the reliability of these dimensions remains modest. Therefore, while the results provide preliminary support for the revised structure, they do not constitute definitive evidence of the scale’s psychometric adequacy. Further research is needed to replicate the factor structure, examine its stability across independent samples, and strengthen evidence for reliability and validity (e.g., convergent and discriminant validity).

### 4.1. Limitations

The present study yielded positive results, but several limitations should be considered. First, the cross-sectional design precludes conclusions about causality and temporal stability. Second, the exclusive reliance on self-report measures may introduce biases, including social desirability and shared method variance. The absence of multi-informant or observational data further limits the scope of validation.

Third, the modified factor structure differs from that of the original instrument, potentially limiting comparability with previous studies. Accordingly, the present findings should be interpreted as evidence for an empirically adapted version rather than a full validation of the original VASQ. In addition, borderline internal consistency in two subscales warrants cautious interpretation.

The use of a convenience sample may also limit generalizability, as participating institutions may not be representative of all RCSs. Furthermore, the identification of cognitive impairments relied on professional judgment rather than on standardized assessment, potentially introducing variability in participant selection.

Finally, some items may not fully capture the relational dynamics of adolescents in residential care and may be susceptible to desirability bias, particularly in contexts where autonomy and privacy are limited (e.g., items 14: “I look forward to spending time on my own,” 15: “I like making decisions on my own,” and 21: “It’s important to have people around me”).

The specific context of residential care should be considered when interpreting the results, and caution is warranted when generalizing these findings to adolescents in other contexts.

Future research should aim to replicate these findings in independent samples, examine longitudinal stability, and use multimethod approaches to assess attachment. The convergent validity of the VASQ could not be evaluated against instruments measuring similar constructs.

Despite these limitations, the study has several strengths, including a national sampling frame, inclusion of youth perspectives on critical developmental issues, and high response rates from a hard-to-reach, under-researched population. Additionally, the observed cutoffs may be useful to other VASQ users who adopt the current factor structure. However, given the variability of RCSs and welfare systems globally, findings should be generalized cautiously to populations outside Portugal, to different care settings, or when comparing the VASQ with other attachment measures. The shorter version of the VASQ remains advantageous, focusing on social aspects and interpersonal approaches to attachment that institutional staff can use to monitor interventions. Because the items are not directed at a particular person, they can be completed by technicians or institutional staff to monitor the effectiveness of the intervention.

### 4.2. Implications for Practice

The findings offer preliminary support for the VASQ’s potential as a brief, accessible tool for assessing attachment-related vulnerabilities in residential care settings [22]. In particular, identifying ambivalent attachment patterns may help practitioners better recognize adolescents at increased risk of psychological maladjustment who could benefit from closer monitoring or targeted relational support [54]. The VASQ may serve as a structured measure to support professional practice [22] without requiring specific training to administer. The VASQ is likely to be a resource for understanding the vulnerabilities faced by young people in the care system and for measuring changes in attachment over time, particularly in response to interventions or significant life transitions [55,56].

However, these results should be interpreted with caution. The present study provides initial validation of a revised factor structure; further research is needed to establish its robustness, generalizability, and clinical utility across diverse samples and contexts. Accordingly, the VASQ should not yet be considered a standalone screening instrument for routine use but rather a complementary tool that may support clinical judgment and ongoing assessment. In light of these limitations, any potential clinical application of the VASQ should be approached cautiously. While the instrument may offer a useful framework for exploring attachment-related vulnerabilities, its current form should not be interpreted as providing a robust or definitive clinical classification. Rather, it may serve as a complementary tool to support clinical judgment, pending further validation and refinement.

The instrument may be particularly useful for informing case conceptualization and guiding relationship-based interventions, especially in institutional settings where attachment disruptions are common. Its brief, accessible format makes it a potentially valuable resource for practitioners, though its use should be complemented by other sources of information.

Strengthening stable, responsive caregiving relationships remains a key priority for promoting emotional regulation and adjustment in this population. Establishing high-quality relationships with care workers can serve as a protective factor [47], enabling youth to process prior experiences of insecure attachment. Consequently, it is essential to provide caregivers with training to build the competencies and attitudes needed to address the unique challenges of working with this population. It is important to recognize that attachment behaviors develop through interaction with a nurturing caregiving environment [7] and are neither inherently positive nor negative; rather, their impact is shaped by developmental experiences and current relational dynamics [51].

In this context, tools such as the revised VASQ may contribute to a more structured understanding of youths’ relational patterns, though further validation is required before recommending broader implementation.

## 5. Conclusions

This study provides preliminary evidence supporting a shortened, restructured version of the VASQ for adolescents in residential care. The findings highlight the potential relevance of this revised structure for understanding attachment-related vulnerabilities in this population and underscore the need for further validation in independent samples and contexts.

The results indicate that insecure-ambivalent attachment is most strongly associated with psychological difficulties, whereas insecure-avoidant attachment shows more domain-specific effects. The proximity-seeking dimension appears to play a more complex, potentially adaptive role.

The proposed version of the instrument demonstrates acceptable psychometric properties and offers preliminary support for its use in residential care settings. Analyses provide meaningful evidence of concurrent validity and practical utility. It should be considered an empirically adapted measure rather than a direct validation of the original VASQ. Further research is needed to replicate these findings, assess the stability of the factor structure, and provide additional evidence of validity across diverse samples and settings.

## Figures and Tables

**Table 1:** Descriptive statistics of VASQ items, item-total correlation, and alpha if the item is deleted.

VASQ Items	M	SD	S	K	Item-Total Correlation	α If Item Deleted
1	3.31	1.18	−0.34	−0.77	0.31	0.83
2	3.06	1.09	−0.23	−0.66	0.13	0.83
3	3.32	1.10	−0.28	−0.47	0.51	0.82
4	3.27	1.21	−0.28	−0.81	0.38	0.82
5	3.40	1.15	−0.36	−0.60	0.50	0.82
6	3.13	1.22	−0.23	−0.82	0.36	0.82
7	3.15	0.98	−0.34	−0.15	0.26	0.83
8	3.03	1.18	−0.02	−0.81	0.51	0.82
9	2.80	1.19	0.10	−0.85	0.43	0.82
10	2.74	1.22	0.19	−0.82	0.57	0.81
11	4.05	1.08	−1.02	0.29	0.33	0.83
12	2.45	1.19	0.48	−0.59	0.28	0.83
13	2.97	1.27	0.07	−1.01	0.46	0.82
14	2.93	1.18	0.04	−0.74	0.34	0.82
15	3.85	1.07	−0.77	0.14	0.18	0.83
16	3.47	1.22	−0.35	−0.81	0.44	0.82
17	2.35	1.19	0.54	−0.66	0.30	0.83
18	3.47	1.16	−0.46	−0.47	0.47	0.82
19	3.09	1.22	−0.07	−0.92	0.52	0.82
20	2.85	1.24	0.14	−0.87	0.54	0.82
21	3.52	1.12	−0.50	−0.28	0.18	0.83
22	3.57	1.19	−0.49	−0.64	0.46	0.82

Note. *M* = Mean; *SD* = Standard deviation; *S* = Skewness; *K* = Kurtosis.

**Table 2:** EFA solution of the VASQ with three factors.

No	Item	Factor			Communalities
		1	2	3	
10	I feel people are against me	0.75			0.62
12	I often get into arguments	0.71			0.55
9	People close to me often get on my nerves	0.67			0.45
20	I feel people haven’t done enough for me	0.63			0.47
17	I feel uneasy when others confide in me	0.54			0.42
8	I feel uncomfortable when people get too close to me	0.51			0.40
14	I look forward to spending time on my own	0.43			0.26
22	I find it difficult to confide in people		0.74		0.57
15	I like making decisions on my own		0.61		0.42
18	I find it hard to trust others		0.58		0.43
1	I take my time getting to know people		0.49		0.25
4	I miss the company of others when I am alone			0.72	0.54
21	It’s important to have people around me			0.64	0.41
7	I usually rely on advice from others when I’ve got a problem			0.61	0.45
6	I worry a lot if people I live with arrive back later than expected			0.58	0.37
2	I rely on others to help me make decisions			0.56	0.39
Explained variance		23%	12%	9%	

Factor labels: 1 = Insecure-ambivalent; 2 = Insecure-avoidant; 3 = Proximity-seeking.

**Table 3:** Goodness-of-Fit Indices for the tested VASQ model using CFA and comparison with previous studies.

Models/Indices	$\chi^{2}/df$	*p* Value	CFI	TLI	RMSEA	RMSEA 90% CI
Present study: 3 factors, 13 items	131/62	<0.001	0.92	0.90	0.06	0.05–0.08
Original study [31]: 2 factors, 22 items	837/208	<0.001	0.74	0.71	0.08	0.08–0.09
Validation study [34]: 4 factors, 14 items	225/71	<0.001	0.89	0.85	0.07	0.06–0.08

*χ*^2^/*df* = Chi-square/degrees of freedom; CFI = Comparative Fit Index; TLI = Tucker–Lewis Fit Index; RMSEA = Root Mean Square Error of Approximation; CI = Confidence interval.

**Table 4:** Cronbach’s alphas, mean inter-item correlations, and corrected item-total correlation ranges.

VASQ Dimensions	Alpha	MIIC	CITCR
Insecure-ambivalent	0.78	0.37	0.36–0.62
Insecure-avoidant	0.66	0.40	0.37–0.54
Proximity-seeking	0.67	0.34	0.38–0.50

Note. Alpha: Cronbach’s alpha; MIIC: mean inter-item correlation; CITCR: corrected item-total correlation range.

**Table 5:** Descriptive statistics and correlations between VASQ dimensions and psychological adjustment (N = 438).

	1	2	3	4	5	6	7	8	9
1. Insecure-ambivalent	-	0.44 ***	0.15 **	0.53 ***	0.41 ***	0.35 ***	0.31 ***	0.40 ***	−0.11 *
2. Insecure-avoidant		-	0.07	0.26 **	0.34 **	0.04	0.09	0.23 **	0.15 *
3. Proximity-seeking			-	0.14 **	0.28 ***	0.02	0.09	−0.04	0.27 ***
4. SDQ total				-	0.75 ***	0.70 ***	0.70 ***	0.63 ***	−0.14 **
5. Emotional problems					-	0.27 ***	0.37 ***	0.37 ***	0.21 ***
6. Behavioral problems						-	0.42 ***	0.33 ***	−0.31 ***
7. Hyperactivity/inattention							-	0.12 *	−0.15 **
8. Peer relationship problems								-	−0.19 ***
9. Prosocial behaviors									-
*M*(*SD*)	16.69	10.34	12.61	15.95	4.63	3.09	4.82	3.41	7.21
	(4.83)	(2.69)	(3.15)	(6.18)	(2.43)	(2.05)	(2.31)	(2.06)	(2.30)

Note. * *p* < 0.05; ** *p* < 0.01; *** *p* < 0.001.

**Table 6:** ROC analysis for VASQ dimensions predicting SDQ domains.

VASQ Dimensions	SDQ Domains	AUC (95% CI)	*p*	Cutoff	Sensitivity	Specificity	Youden Index (*J*)
Insecure-ambivalent	Emotional symptoms	0.707 0.647–0.768	0.000	19	0.561	0.756	0.317
	Behavioral problems	0.689 0.631–0.746	0.000	18	0.625	0.659	0.284
	Hyperactivity	0.633 0.569–0.697	0.000	16	0.779	0.429	0.208
	Peer relationship	0.656 0.582–0.729	0.000	17	0.733	0.519	0.252
	SDQ Total score	0.791 0.745–0.837	0.000	18	0.706	0.725	0.431
Insecure-avoidant	Emotional symptoms	0.646 0.579–0.713	0.000	13	0.418	0.826	0.245
	Peer relationships	0.644 0.568–0.720	0.000	12	0.533	0.693	0.226
	SDQ Total score	0.618 0.561–0.674	0.000	12	0.471	0.722	0.192
Proximity-seeking	Emotional symptoms	0.618 0.550–0.685	0.001	15	0.418	0.779	0.198
	SDQ Total score	0.567 0.508–0.625	0.025	15	0.338	0.768	0.106

Note. AUC = Area under the curve; Hyperactivity = hyperactivity/inattention; Peer relationship = peer relationship problems.

**Table 7:** Associations between VASQ dimensions and SDQ subscales and total score.

		Emotional	Behavioral	Hyperactivity	Peer	Prosocial	SDQ Total
Insecure-ambivalent	χ^2^ (*df* = 1)/*p*	23.87/<0.001	26.41/<0.001	12.81/<0.001	5.75/0.017	0.20/0.655	72.10/<0.001
	Cramer’s V/*p*	0.23/<0.001	0.25/<0.001	0.17/<0.001	0.12/0.017	0.02/0.655	0.41/<0.001
	OR 95% CI	0.32 [0.20–0.51]	0.31 [0.20–0.49]	0.43 [0.27–0.69]	0.52 [0.30–0.89]	0.88 [0.50–1.56]	0.16 [0.10–0.25]
Insecure-avoidant	χ^2^ (*df* = 1)/*p*	16.75/<0.001	0.20/0.659	1.44/0.230	11.87/<0.001	1.95/0.162	15.52/<0.001
	Cramer’s V/*p*	0.20/<0.001	0.02/0.659	0.06/0.230	0.17/<0.001	0.07/0.162	0.19/<0.001
	OR 95% CI	0.39 [0.25–0.62]	0.90 [0.57–1.43]	0.75 [0.47–1.20]	0.39 [0.22–0.67]	1.56 [0.83–3.03]	0.43 [0.28–0.66]
Proximity-seeking	χ^2^ (*df* = 1)/*p*	15.28/<0.001	0.14/0.711	5.40/0.020	0.12/0.727	7.84/0.005	5.46/0.019
	Cramer’s V/*p*	0.19/<0.001	0.02/0.711	0.11/0.020	0.02/0.727	0.13/0.005	0.11/0.019
	OR 95% CI	0.39 [0.24–0.63]	0.91 [0.56–1.49]	0.56 [0.35–0.92]	0.90 [0.49–1.64]	3.33 [1.37–7.69]	0.59 [0.38–0.92]

Note. Emotional = emotional problems; Behavioral = behavioral problems; Hyperactivity = Hyperactivity/Inattention; Peer = peer relationship problems; Prosocial = prosocial behaviors; SDQ Total = SDQ total score; df = degrees of freedom; OR = *Odds Ratios*; CI = confidence interval. Outcome variables were coded such that the reference category corresponds to the non-clinical range; therefore, OR values below 1.00 indicate an increased likelihood of clinically significant difficulties, whereas OR values above 1.00 indicate a greater likelihood of non-clinical functioning.

## Data Availability

The data supporting the findings of this study are available from the corresponding author upon reasonable request.

## Abbreviations

The following abbreviations are used in this manuscript:

CFA	Confirmatory Factor Analysis
EFA	Exploratory Factor Analysis
RCS	Residential care setting
SDQ	Strengths and Difficulties Questionnaire
VASQ	Vulnerable Attachment Style Questionnaire

## Appendix A

**Table A1:** ROC analysis for VASQ dimensions predicting SDQ outcomes.

VASQ Dimensions	SDQ Domains	AUC (95% CI)	*p*	Cutoff	Sensitivity	Specificity	Youden Index (*J*)
Insecure-ambivalent	Emotional symptoms	0.707 0.647–0.768	0.000	19	0.561	0.756	0.317
	Behavioral problems	0.689 0.631–0.746	0.000	18	0.625	0.659	0.284
	Hyperactivity	0.633 0.569–0.697	0.000	16	0.779	0.429	0.208
	Peer relationship	0.656 0.582–0.729	0.000	17	0.733	0.519	0.252
	Prosocial behaviors	0.541 0.460–0.622	0.323	17	0.582	0.493	0.075
	SDQ Total score	0.791 0.745–0.837	0.000	18	0.706	0.725	0.431
Insecure-avoidant	Emotional symptoms	0.646 0.579–0.713	0.000	13	0.418	0.826	0.245
	Behavioral problems	0.518 0.458–0.579	0.555	8	0.904	0.168	0.072
	Hyperactivity	0.550 0.483–0.616	0.143	10	0.705	0.385	0.090
	Peer relationships	0.644 0.568–0.720	0.000	12	0.533	0.693	0.226
	Prosocial behaviors	0.417 0.334–0.500	0.050	16	0	1	0.000
	SDQ Total score	0.618 0.561–0.674	0.000	12	0.471	0.722	0.192
Proximity-seeking	Emotional symptoms	0.618 0.550–0.685	0.001	15	0.418	0.779	0.198
	Behavioral problems	0.516 0.452–0.580	0.630	18	0.096	0.952	0.048
	Hyperactivity	0.582 0.515–0.648	0.016	14	0.474	0.656	0.130
	Peer relationships	0.496 0.411–0.581	0.928	17	0.167	0.899	0.066
	Prosocial behaviors	0.314 0.238–0.390	0.000	20	0.036	0.99	0.026
	SDQ Total score	0.567 0.508–0.625	0.025	15	0.338	0.768	0.106

Note. Hyperactivity = hyperactivity/inattention; Peer relationship = peer relationship problems.
